# Birth weight and bone mineral density at 18–19 years: birth cohort 1997–1998

**DOI:** 10.11606/s1518-8787.2023057004179

**Published:** 2023-03-15

**Authors:** Allanne Pereira Araújo, Janaina Maiana Abreu Barbosa, Carolina Abreu de Carvalho, Poliana Cristina de Almeida Fonseca Viola, Cecilia Claudia Costa Ribeiro, Rosangela Fernandes Lucena Batista, Vanda Maria Ferreira Simões

**Affiliations:** I Universidade Federal do Maranhão Programa de Pós Graduação em Saúde Coletiva São Luís MA Brasil Universidade Federal do Maranhão. Programa de Pós Graduação em Saúde Coletiva. São Luís, MA, Brasil; II Universidade Federal do Maranhão Departamento de Saúde Pública São Luís MA Brasil Universidade Federal do Maranhão. Departamento de Saúde Pública. São Luís, MA, Brasil; III Universidade Federal do Piauí Departamento de Nutrição Teresina PI Brasil Universidade Federal do Piauí. Departamento de Nutrição. Teresina, PI, Brasil

**Keywords:** Adolescent, Bone Density, Birth Weight, Cohort Studies

## Abstract

**OBJECTIVE:**

To analyze the association between birth weight and bone mineral density (BMD) in adolescence.

**METHODS:**

A birth cohort study in São Luís, Maranhão, using data from two moments: at birth and at 18–19 years. Exposure was the birth weight in grams, continuously analyzed. The outcome was BMD, using the Z-score index (whole body) measured by double X-ray densitometry (Dexa). A theoretical model was constructed in acyclic graphs to identify the minimum set of adjustment variables – household income, the mother knowing how to read and write at the time of birth, prenatal care, tobacco use during pregnancy, and parity — to evaluate the association between birth weight and bone mineral density in adolescence. Multiple linear regression was used in Stata 14.0 software. A 5% significance level was adopted.

**RESULTS:**

From 2,112 adolescents, 8.2% had low birth weight and 2.8% had a low BMD for their age. The mean full-body Z-score was 0.19 (± 1.00). The highest birth weight was directly and linearly associated with BMD values in adolescence (Coef.: 0.10; 95%CI: 0.02–0.18), even after adjustment for the variables household income (Coef.: -0.33; 95%CI: -0.66–0.33) and the mother knowing how to read and write (Coef.: 0.23%; 95%CI: 0.03–0.43).

**CONCLUSION:**

Although after adjusting the variables the association attenuated, birth weight positively and linearly relates to BMD in adolescence.

## INTRODUCTION

Bone mineral content (BMC) is the amount in kilograms of bone that, divided by its size, results in bone mineral density (BMD) in g/cm^1 , 2^. BMD has been widely studied in adults and the older population, phases in which higher prevalence of osteoporosis and fractures resulting from bone mineral loss occur. However, the bone mineral density achieved in adulthood depends on the peak bone mass acquired up to 20 years old, which makes investigation in adolescents important. Authors believe that an early loss of BMD in adolescence may associate with some pathologies and/or unhealthy lifestyle (diet, physical activity and excessive alcohol consumption)^2 , 3^.

Bone mineralization begins in intrauterine life and extends from childhood to early adulthood, but during childhood and adolescence occurs the greatest growth and development of bone mineral tissue^4 , 5^.

Early life factors, such as birth weight and intrauterine growth restriction, may influence bone mineral mass and the risk of osteoporosis^6^.

Bone health at different stages of life may reflect previous stages, although disagreements on how this process happens exist. Authors believe that insufficient fetal nutrition can lead to permanent changes in the development of the neuroendocrine system — insulin-like growth factor I and growth hormone, influencing bone development throughout life^7^.

In a Norwegian cohort study, newborns with low birth weight presented lower peaks of bone mass and higher frequency of osteoporosis, implying a higher risk of fracture in adulthood^8^.

A systematic review found that higher birth weight is determinant for better bone health, confirming that bone mass programming could exist. The effect may occur in children, but is inconclusive among adolescents. The effect of birth weight on bone mass among children was on BMD and BMC^6^.

The exploration in population-based longitudinal studies on the effect of early life variables and their determination on bone mineral density in adolescence is scarce. However, the study of BMD in younger individuals allows to know the early determinants of bone health, to assist in the planning of individual and collective interventions, reducing the effects of osteoporosis in adults and the older population.

This study aims to analyze the association between birth weight and BMD of adolescents from a birth cohort conducted in São Luís – MA.

## METHODS

### Study Design

This is a longitudinal study with data from a birth cohort conducted in São Luís – MA, Brazil, included in the consortium of RPS cohorts (Ribeirão Preto, Pelotas and São Luís), entitled *Determinants throughout the life cycle of obesity, precursors of chronic diseases, human capital and mental health: a contribution of the Brazilian birth cohorts to the SUS* , developed by the Federal University of Maranhão (UFMA), Ribeirão Preto Medical School (FMRP-USP) and the Federal University of Pelotas (UFPel). This cohort included live newborns of hospital birth of mothers living in the city of São Luís, between March 1997 and February 1998. Participants were evaluated in three phases of life: at birth, in childhood (7–9 years) and in adolescence (18–19 years). For this work, data from the first and third phases were used.

### Study Population and Sampling

The birth cohort was conducted in ten public and private hospitals that provided delivery care from 1997–1998. Systematic sampling was used with stratification proportional to the number of births in each hospital. Thus, one in each seven deliveries were recruited in each hospital unit. In this phase of the cohort, 2,541 live births, stillbirths, single births and multiple deliveries of women living in São Luís participated. We did not include births that occurred outside hospitals and those that occurred in hospitals where there were less than 100 deliveries per year.

The target population included 96.3% of all deliveries in São Luís. Losses due to refusal or impossibility of locating the mother in hospitals occurred in 5.8% of the cases. Excluding multiple and stillborn deliveries, the final sample of this phase corresponded to 2,493 births^9^.

In the third phase, data collection was performed in 2016. To locate the participants, search procedures were used in school and university enrollments, in the addresses and telephone contacts recorded in the first and second phase of the cohort, in the records of military enlistment, and in social media. From these search strategies, 687 attended data collection.

To increase the sample size of the study and prevent future losses, it was decided to include other adolescents born in the city of São Luís between 1997–1998 who had been excluded in the original sample of the cohort at the time of birth, and a draw was made in the SINASC database (information system on live births). A second strategy was the inclusion of adolescent volunteers identified in schools, universities and social media. Data on the birth of these adolescents were answered by the mother. The following criteria were taken into account for the registration: being born in maternity, in the city of São Luís, in 1997.

From this listing, a random draw was made, obtaining a total of 4,593 born in 1997, in the city of São Luís. Of this total, it was possible to contact 1,716, to which all questionnaires were applied. In a second stage, volunteers were identified in schools, universities and social media, thus, contacting 110 adolescents. The volunteers were submitted to the same tests and questionnaires as the other participants of the original cohort.

By these strategies to include new participants, 1,826 adolescents were added to the research from the third phase of this cohort, which was composed of 2,515 adolescents, from the original and those included from this phase^10^.

Thus, adolescents born in São Luís – MA, between March 1997 and February 1998, who presented information about socioeconomic and demographic data, birth and their bone health were included. Pregnant adolescents were excluded, as they could not be submitted to bone densitometry examination, since the equipment emits a small amount of radiation during the evaluation, which totaled 2,112 adolescents for this study.

### Data Collection Procedures and Variables

Data collection of the third phase occurred in 2016. Questionnaires and body composition assessment tests were completed by duly trained health professionals. The information was recorded in the Research Electronic Data Capture (Redcap^®^) program, which is online and secure for the registration and storage of research data^11^.

To verify possible selection bias, the variables gender and the mother knowing how to read and write at the time of birth were compared between the members of the original cohort and the adolescents included in the study, where differences could be observed in opposite directions.

Follow-up losses were higher for women (79%) when compared to men (71.5%, p < 0.001), and for those with 0–4 years of study (80.2%) compared to those with more than 12 years of study (74.8%, p = 0.020). However, the opening of the cohort for the insertion of adolescents brought greater participation of women with lower schooling^10 , 12^.

### Variables

The main explanatory independent variable was birth weight, used continuously. For individuals who participated in the cohort since birth, this variable was collected via medical records in maternity hospitals, and for those included in the third phase, it was self-reported by the mothers and confirmed by the SINASC database.

BMD, which is the relationship between the BMC and the area of the evaluated bone, expressed in g/cm^2^, was considered the dependent variable of the study. The evaluation of the bone mineral density of adolescents was performed by means of double X-ray densitometry (Dexa), based on enCORE and Lunar Prodigy model of the brand GE Healthcare^®^. The exam is safe and takes about 15 minutes to scan the entire body. The adolescent was asked to lie down at the table in supine position and remain motionless during the scan. The teenager wore light clothes, from lycra, tight to the body, typically a short shorts, for men and women, and a “top”, for women. They were barefoot, without dentures and other types of metallic materials.

The Z-score calculated by the difference between the BMD and the mean population of the same age, gender, and ethnicity was considered. Total body measurement was considered as one of the most sensitive sites to evaluate bone mineral density because it is more associated with a higher risk of fractures and bone diseases in this population^14^. To determine low rates, BMD was categorized into normal when greater than or equal to −2 standard deviations, and low when less than −2 standard deviations^13^. Bone mineral density was used as a continuous variable in the analyses.

The maternal data used were all related to the period of birth: maternal age (< 20 years, 20–34 years and ≥ 35 years), knowing how to read and write (no/yes), marital status (with/without a partner), parity (one delivery, two deliveries and ≥ three deliveries) and household income (up to one minimum wage, two minimum wages and ≥ three minimum wages), tobacco use during pregnancy (no/yes), number of medical consultations during prenatal care (< 6 consultations and ≥ 6 consultations), and type of delivery (vaginal/cesarean).

Data from adolescents: gender (male/female), age (18–19 years), maternal education (elementary school, high school, technical-high school, incomplete higher education, and education for young people and adults), marital status (with/without a partner), economic class according to the Criteria of Economic Classification in Brazil (ABEP, 2015), and gainful employment (yes/no).

### Theoretical Model of the Association Between Birth Weight and BMD in Adolescence

Directed acyclic graphs (DAGs) are causal diagrams used to select variables, with the objective of controlling confounding and avoiding unnecessary adjustments. A theoretical model was constructed, based on the literature, to analyze the association between birth weight and BMD in adolescence ( Figure ), using the browser *Dagitty* ^14^.

**Figure:**
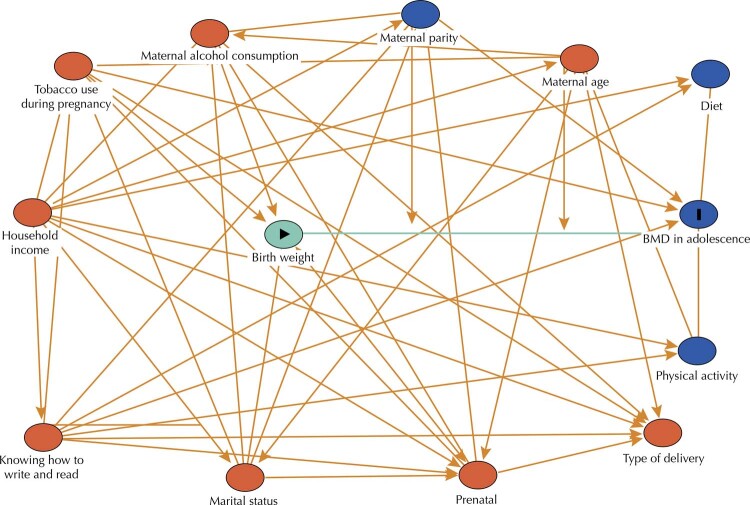
Directed acyclic graph on the association of birth weight and bone mineral density (BMD) in adolescents. The directed acyclic graph (DAG) was constructed based on the theoretical models of Martínez-Mesa^6^ and Baird^20^ for bone mass prediction. The diagram uses a set of arrows to characterize causal associations between exposure and outcome and, in addition, to identify relationships between variables that influence exposure or outcome. In this type of chart, the causes are called ancestors. Thus, the DAG allowed the selection of an appropriate set of confounding variables, as well as identifying variables of collider in a non-causal or polarization pathway, to be retained within model^15^. The vertex (circle) in yellow indicates exposure, while the one in blue with black outline indicates the outcome. The other vertices in red are ancestors of the exhibition and those in blue, ancestors of the outcome. The edges (arrows) of the polarization paths are highlighted in red and that of the causal path in green.

In the analysis of the DAGs, two models were suggested: one of total effect that excludes the mediating variables, suggesting adjustment for maternal age, marital status, parity, household income, prenatal care, tobacco use, type of delivery, the mother knowing how to read and write at the time of birth. The other model had direct effect, in which the mediating variables are added, seeking to decompose an unmediated effect. In this model, we analyzed the minimum set of variables: parity, household income, prenatal care, tobacco use, and the mother knowing how to read and write at the time of birth.

### Statistical Analysis

The data were exported from REDCap to be analyzed in the statistical program Stata^®^ version 14.0. To verify whether the study variables followed the normal distribution pattern, distribution graphs (histograms) were constructed and the Shapiro Wilk test was applied. Categorical variables were described by absolute and relative frequencies. The continuous variables were described by mean and standard deviation.

Multiple linear regression analysis was used to test the association between birth weight and BMD in adolescence. Residue analysis was also performed to check the assumptions of the linear regression model and identification of outliers *.*

Significance level was set at 5%. The regression coefficient (Coef.) and the respective confidence intervals (95%CI) were estimated.

### Ethical Aspects

The study was approved by the Research Ethics Committee of the Hospital das Clínicas of the Ribeirão Preto Medical School (No. 28/2004 and No. 10073/2009) and the Research Ethics Committee of the University Hospital/UFMA (No. 3104-476/2005). In 2015, it was also approved by the Research Ethics Committee of the University Hospital/UFMA (No. 19/2015). All participants signed the informed consent form.

## RESULTS

The adolescents of the cohort in São Luís had a prevalence of 2.8% for low bone mass for chronological age and the mean full-body Z-score was 0.19 (± 1.00). In the sample of 2,112 adolescents, 95.1% of mothers did not use tobacco during pregnancy, 68.4% were in the age group of 20–34 years, 95% could read and write, 77.5% lived with a partner, 49.6% were primiparous, and 87% had a household income of up to one minimum wage at the time of birth. Regarding prenatal care, 56% had six or more medical consultations, 63.9% had vaginal delivery, and 8.2% of the newborns had low birth weight ( Table 1 ).

**Table 1:** Socioeconomic and demographic characteristics of mothers and their newborns in the first phase of the RPS birth cohort. São Luís, MA, Brazil, 1997–1998.

Variables	n	%
Age (years old)		
< 20	574	27.2
20–34	1,444	68.4
≥ 35	94	4.4
Knowing how to read and write		
Yes	2,009	95.0
No	103	5.0
Marital status		
With partner	1,637	77.5
Without a partner	475	22.5
Parity (deliveries)		
1	1,047	49.6
2	629	29.8
≥ 3	436	20.6
Household income (MW)		
≤ 1	1,838	87.0
2	171	8.1
≥ 3	103	4.9
Tobacco use		
No	2,007	95.1
Yes	105	4.9
Number of medical consultations during the PC		
< 6	929	44.0
≥ 6	1,183	56.0
Type of delivery		
Vaginal	1,350	63.9
Cesarean section	762	36.1
Sex of NB		
Male	1,008	47.7
Female	1,104	52.3
LBW		
No	1,939	91.8
Yes	173	8.2

Total	2,112	100

MW: minimum wage; PC: prenatal care; NB: newborn; LBW: low birth weight.

Female adolescents (52.3%), 18 years old (73.7%), single (96.8%), belonging to economic class C (76.2%), without gainful employment (60.8%), and attending high school (34.1%) prevailed ( Table 2 ).

**Table 2:** Socioeconomic and demographic characteristics of adolescents, in the third phase of the RPS birth cohort, São Luís, MA, Brazil, 2016–2017.

Variables	n	%
Sex		
Male	1,008	47.7
Female	1,104	52.3
Age (years old)		
18	1,599	73.7
19	513	26.3
Maternal schooling		
Elementary School	7	0.3
High School	720	34.1
Mid-level technical course	125	6
Incomplete higher education	536	25.4
EJA	46	2.2
Ignored	678	32
Marital status		
Without a partner	2,045	96.8
With partner	67	3.2
Economy class^a^		
A/B	264	12.5
C	1,056	76.2
D/E	233	11.0
Ignored	11	0.3
Gainful Employment		
Yes	828	39.2
No	1,284	60.8
BMD		
Normal	2,053	97.2
Low	59	2.8

Total	2,112	100

EJA: Education for Young People and Adults; BMD: bone mineral density.

^a^ According to ABEP (2015).

Birth weight was associated with BMD in adolescence (Coef.: (0.10%; 95%CI: 0.02–0.18). This effect of birth weight was attenuated in the analysis of the model adjusted for the confounders (p = 0.014; Coef.: (0.10%; 95%CI: 0.02–0.18). However, in this direct effect model, the highest household income (p = 0.030; Coef.: −0.33; 95%CI: -0.66–0.33) and the mother knowing how to read and write (p = 0.024; Coef.: 0.23; 95%CI: 0.03–0.43) remained associated with the outcome ( Table 3 ).

**Table 3:** Adjusted analysis of the total effect and direct effect models of factors associated with birth weight with bone mineral density (BMD) in adolescence of the RPS birth cohort. São Luís, MA, Brazil, 2016–2017.

Variables	BMD in adolescence					

	Full effect model			Direct effect model		
	
	Coef.	95%CI	p	Coef.	95%CI	p
Maternal age (years old)						
< 20	-0.05	-0.15 to 0.05	0.353	-	-	-
20–34	1	-	1	-	-	-
≥ 35	0.03	-0.20 to 0.26	0.772	-	-	-
Marital status	0.04	-0.14 to 0.06	0.453	-	-	-
Parity (deliveries)						
2	0.03	-0.07 to 0.13	0.599	0.03	-0.07 to 0.13	0.599
≥ 3	-0.08	-0.21 to 0.04	0.178	-0.08	-0.21 to 0.04	0.180
Household income (MW)						
2	-0.08	-0.57 to 0.40	0.736	0.10	-0.17 to 0.36	0.480
≥ 3	-0.48	-0.85 to 0.10	**0.012**	-0.33	-0.66 to 0.33	**0.030**
Realization of PC	-0.01	-0.10 to 0.08	0.871	-0.01	-0.01 to 0.08	0.844
Tobacco use	-0.06	-0.25 to 0.14	0.570	-0.05	-0.25 to 0.14	0.583
Type of delivery	0.01	-0.08 to 0.11	0.819	-	-	**-**
Knowing how to read and write	0.49	0.10 to 0.91	**0.017**	0.23	0.03 to 0.43	**0.024**
Birth weight	0.10	0.02 to 0.18	**0.012**	0.10	0.02 to 0.18	**0.014**

Coef.: regression coefficient; 95%CI: 95% confidence interval; MW: minimum wage; PC: prenatal care. Total effect model: adjusted for maternal age, marital status, parity, household income, PC, tobacco use, type of delivery, knowing how to read and write, and birth weight.

Total effect model: adjusted for maternal age, marital status, parity, household income, PC, tobacco use, type of delivery, knowing how to read and write, and birth weight. Only the variables with p-value < 0.05 were considered statistically significant.

In the model for total effect (p = 0.012; Coef.: 0.10; 95%CI: 0.02–0.18) and direct (p = 0.014; Coef.: 0.10; 95%CI: 0.02–0.18) we observed a positive linear association between birth weight and bone mass in adolescence, even after adjusting for the variables family income (p = 0.030; Coef.: −0.33; 95%CI: -0.66–0.33) and mothers knowing how to read and write (p = 0.024; Coef.: 0.23%; 95%CI: 0.03–0.43).

We also observed a linear relationship between birth weight and bone mass in adolescence: as birth weight (p = 0.012; Coef.: 0.10; 95%CI: 0.02–0.18) increases by 1 kg, bone mass in adolescence also increases by 0.10 g/cm^2^. Therefore, the higher the value of birth weight, the higher the value of BMD achieved in adolescence ( Table 3 ).

## DISCUSSION

In this study, as birth weight increases, the BMD values in adolescence also increase, and we verified an independent association of the fit for household income and the mother knowing how to read and to write at the time of birth. By adjusting the direct effect model for the variables mentioned the magnitude of this association decreased.

Few adolescents presented low bone mineral density (2.8%). Nevertheless, these values indicate previous bone mass impairment, because at 18 and 19 years old bone mass peaks, when it is expected to be at normal values. These findings are important for the investigation of factors, such as fetal development, birth weight, genetic factors — the main determinants of peak bone mass in adulthood — and modifiable factors (diet, physical activity, and tobacco use), which are causing a lower BMD early^15^.

We observed a relevant finding: as birth weight increases by 1 kg, BMD in adolescence also increases by 0.10 g/cm^2^. The fact that the health status of the skeleton, in the different stages of life, is a reflection of previous stages explains this association. Prospective studies show that low birth weight relates to the development of chronic diseases in adulthood, according to the theory Developmental Origins of Health and Disease (DOHaD)^6^.

Although how this process happens is still at issue, one of the possible explanations for this association would be the hormones GH (growth hormone) with cortisol as one of the determinants of bone loss^16^, ratifying the theory that environmental stressors during intrauterine or early postnatal life cause changes in the sensitivity of growth plaque to GH and cortisol, reducing the size of the skeleton, which may lead to a decrease in mineralization, and predispose to an accelerated rate of bone loss during adulthood^17^.

Birth weight is an important determinant of the peak bone mass reached in adolescence and one of the main factors for maintaining adequate levels of bone mass in adulthood. Despite the existence of some gaps in the mechanisms that involve this association, some studies corroborate the hypothesis that osteoporosis can be programmed at the beginning of development^6 , 18 , 19^. According to Baird et al.^19^, this programming can be classified into two studies: 1) those who explored the association between the physiological system of individuals who may have been “programmed” and the rate of bone mass loss due to aging; 2) studies that investigated the influence of the constitution of the body, nutrition, and lifestyle of mothers on the bone mass of their offspring.

A systematic review^20^ corroborated our findings by showing that birth weight is associated with BMD, contributing to the knowledge that this association is also observed in adolescents. This is an important finding for public health policies, since a reduction in bone mineral density strongly associates with an increased risk of fractures^20^.

The fact that the mother can read and write at the time of birth and has a household income of at least three minimum wages had a direct effect on the association studied here. Individuals exposed to favorable socioeconomic conditions and, consequently, healthier lifestyle seem to develop their potential for adequate BMD better.

Mothers living in unfavorable socioeconomic conditions are more likely to conceive newborns with lower birth weight^21^. The insufficient supply of nutrients to the fetus (due to social vulnerability) may lead to adaptations of cells and their metabolism^22^ and, therefore, influence baseline levels of GH and cortisol in adolescence, because they are involved in obtaining peak bone mass and predispose to greater bone loss during adulthood^23 , 24^.

From the clinical point of view, considering that bone loss is a natural and irreversible process that occurs with aging, one of the best strategies for the prevention of osteoporosis is to optimize the peak bone mass of an individual^25^. From the epidemiological point of view, the earlier a preventive procedure begins, the better the chance of achieving desirable results^20^.

A study in Amsterdam showed a positive association between birth weight and whole body and hip BMD at 36 years old. The effects of birth weight on bone mineral density (total, lumbar spine, and femoral neck) are maintained until advanced ages, above 70 years^25^.

In a study conducted by Martínez-Mesa et al.^6^, birth weight was the highest criterion of BMD. These results reinforce the hypothesis raised in this study that adequate birth weight may be important to maximize bone mass early, especially in adolescence^15 , 26^.

Thus, low values of birth weight would act as risk markers for future reduced bone mineral density, since this situation is characterized by low bone mass and deterioration of bone tissue microarchitecture, with subsequent increase in fragility and susceptibility to fractures^7^.

This study presented a limitation: the loss of participants during follow-up, especially in the third phase, due to difficulties in finding adolescents, despite the use of several search strategies. Such losses may have contributed to underestimate the associations that these strata prevailed more. The mothers of the adolescents included in the study reported their birth weights, however, we confirmed the numbers in SINASC, minimizing the possibility of memory bias.

The adjustment of the proposed model excluded the food intake and the practice of physical activity of the adolescents, because DAG did not identified these variables.

Highlights of the study: the longitudinal design of this study, the causal theoretical model based on DAG for the identification of the minimum set of variables necessary for adjustment to study the causal effect of birth weight on BMD. The evaluation of bone mineral density using DEXA, which is considered gold standard in adolescence, is also an important highlight of the study. Moreover, BMD is an important determinant of bone health at older ages, and this early evaluation, even in adolescence, was possible in this study.

Although osteoporosis is a more prevalent disease in the older population, it should be prevented to promote bone health during childhood and adolescence, ensuring that BMD reaches its optimal peak and develops properly.

The performance of studies that clarify and point to empirical understanding about the process of bone mass acquisition during intrauterine life, childhood, and adolescence until reaching the bone peak are of great relevance, clarifying the need for performances of studies in earlier stages.
