# Supplementary material for: Birth weight and bone mineral density at 18–19 years: birth cohort 1997–1998
Source: Rev Saude Publica. 2023 Mar 15;57:9. doi: 10.11606/s1518-8787.2023057004179 (PMC10118403; doi:10.11606/s1518-8787.2023057004179)
Supplement: Translation

# Translation: Peso ao nascer e densidade mineral óssea aos 18–19 anos: coorte de nascimentos 1997–1998

**Authors:** Allanne Pereira Araújo, Janaina Maiana Abreu Barbosa, Carolina Abreu de Carvalho, Poliana Cristina de Almeida Fonseca Viola, Cecilia Claudia Costa Ribeiro, Rosangela Fernandes Lucena Batista, Vanda Maria Ferreira Simões

**Affiliations:** I Brasil Universidade Federal do Maranhão. Programa de Pós Graduação em Saúde Coletiva. São Luís, MA, Brasil; II Brasil Universidade Federal do Maranhão. Departamento de Saúde Pública. São Luís, MA, Brasil; III Brasil Universidade Federal do Piauí. Departamento de Nutrição. Teresina, PI, Brasil

**Keywords:** Adolescente, Densidade Óssea, Peso ao Nascer, Estudos de Coortes

## Abstract

**OBJETIVO:**

Analisar a associação entre o peso ao nascer e a densidade mineral óssea (DMO) na adolescência.

**MÉTODOS:**

Estudo de coorte de nascimentos em São Luís, Maranhão, utilizando dados de dois momentos: ao nascimento e aos 18–19 anos. A exposição foi o peso ao nascer em gramas, analisado de forma contínua. O desfecho foi a DMO, utilizando o índice Z-escore (corpo inteiro) medido pela densitometria por dupla emissão de raios X (DEXA). Foi construído modelo teórico em gráficos acíclicos direcionados para identificar o conjunto mínimo de variáveis de ajuste – renda familiar, a mãe saber ler e escrever à época do nascimento, realização de pré-natal, tabagismo durante a gestação e paridade – para avaliar a associação entre o peso ao nascer e a densidade mineral óssea na adolescência. Utilizou-se regressão linear múltipla no *software* Stata 14.0. O nível de significância adotado foi de 5%.

**RESULTADOS:**

Dos 2.112 adolescentes, 8,2% apresentaram baixo peso ao nascer e 2,8% apresentaram DMO considerada baixa para a idade. O Z-escore médio de corpo inteiro foi de 0,19 (± 1,00). O maior peso ao nascer foi associado de forma linear e direta aos valores de DMO na adolescência (Coef.: 0,10; IC95% 0,02–0,18), mesmo após ajuste para as variáveis renda familiar (Coef.: -0,33; IC95% -0,66–0,33) e a mãe saber ler e escrever (Coef.: 0,23; IC95% 0,03–0,43).

**CONCLUSÕES:**

Apesar de a associação ter sido atenuada após ajuste das variáveis, o peso ao nascer está associado de forma positiva e linear à DMO na adolescência.

## INTRODUÇÃO

O conteúdo mineral ósseo (CMO) é definido pela quantidade em quilogramas de osso que, dividido pelo seu tamanho, resulta na densidade mineral óssea (DMO) mensurada em g/cm^1 , 2^. A DMO tem sido amplamente estudada em adultos e idosos, fases em que ocorrem maiores prevalências de osteoporose e fraturas decorrentes da perda mineral óssea. Entretanto, a densidade mineral óssea alcançada na vida adulta depende do pico de massa óssea adquirido até os 20 anos de idade, o que torna importante a investigação em adolescentes. Acredita-se que uma perda precoce da DMO na adolescência pode estar associada a algumas patologias e/ou ao estilo de vida não saudável (alimentação, atividade física e consumo excessivo de álcool)^2 , 3^.

A mineralização óssea tem seu início na vida intrauterina e estende-se da infância até o início da idade adulta, mas é durante a infância e adolescência que ocorre o maior crescimento e desenvolvimento do tecido mineral ósseo^4 , 5^.

A massa mineral óssea e o risco de osteoporose podem ser influenciados por fatores do início da vida, como o peso ao nascer e restrição do crescimento intrauterino^6^.

A saúde óssea nas diferentes etapas da vida aparenta ser um reflexo de etapas anteriores, embora não exista um consenso de como este processo acontece. Acredita-se que a nutrição fetal insuficiente pode levar a mudanças permanentes no desenvolvimento do sistema neuroendócrino – fator de crescimento semelhante à insulina I e hormônio do crescimento – influenciando no desenvolvimento ósseo ao longo da vida^7^.

Em estudo de coorte norueguês, recém-nascidos com baixo peso ao nascer apresentaram picos mais baixos de massa óssea e maior frequência de osteoporose, implicando em risco maior de fratura na vida adulta^8^.

Uma revisão sistemática constatou que o peso mais elevado ao nascer é determinante para uma melhor saúde óssea, ratificando que poderia haver programação de massa óssea. O efeito pode ocorrer em crianças, mas é inconclusivo entre adolescentes. O efeito do peso ao nascer sobre a massa óssea entre as crianças foi na DMO e no CMO^6^.

O efeito de variáveis precoces da vida e sua determinação sobre a densidade mineral óssea na adolescência têm sido pouco explorados em estudos longitudinais de base populacional. Contudo, o estudo da DMO em indivíduos mais jovens permite conhecer os determinantes precoces da saúde óssea, a fim de auxiliar no planejamento de intervenções individuais e coletivas para diminuir os efeitos da osteoporose no indivíduo adulto/idoso.

Este estudo pretende analisar a associação entre o peso ao nascer e a DMO de adolescentes pertencentes a uma coorte de nascimentos realizada em São Luís – MA.

## MÉTODOS

### Delineamento do Estudo

Trata-se de um estudo longitudinal com dados de uma coorte de nascimentos realizada em São Luís – MA, Brasil, incluída no consórcio de coortes RPS (Ribeirão Preto, Pelotas e São Luís), intitulado *Determinantes ao longo do ciclo vital da obesidade, precursores de doenças crônicas, capital humano e saúde mental: uma contribuição das coortes de nascimentos brasileiras para o SUS* , desenvolvida pela Universidade Federal do Maranhão (UFMA), Faculdade de Medicina de Ribeirão Preto (FMRP-USP) e Universidade Federal de Pelotas (UFPel). Esta coorte incluiu recém-nascidos vivos de parto hospitalar de mães residentes no município de São Luís, entre março de 1997 e fevereiro de 1998. Os participantes foram avaliados em três fases da vida: ao nascimento, na infância (7–9 anos) e na adolescência (18–19 anos). Para este trabalho foram usados os dados da primeira e terceira fases.

### População do Estudo e Amostragem

A coorte dos nascimentos foi conduzida em 10 hospitais públicos e privados que forneciam assistência ao parto, no período de 1997–1998. Utilizou-se amostragem sistemática com estratificação proporcional ao número de nascimentos em cada hospital. Assim, foram recrutados um em cada sete partos em cada unidade hospitalar. Nesta fase da coorte participaram 2.541 nascidos vivos, natimortos, partos únicos e partos múltiplos de mulheres residentes em São Luís. Não foram incluídos os nascimentos que não ocorreram nos hospitais e os ocorridos em hospitais onde havia menos de 100 partos por ano.

A população-alvo contemplou 96,3% de todos os partos ocorridos em São Luís. As perdas devido à recusa ou impossibilidade de localizar a mãe nos hospitais ocorreram em 5,8% dos casos. Excluindo-se partos múltiplos e natimortos, a amostra final desta fase correspondeu a 2.493 nascimentos^9^.

Na terceira fase, a coleta de dados foi realizada no ano de 2016. Para a localização dos participantes, foram utilizados procedimentos de busca em matrículas escolares e de universidades, nos endereços e contatos telefônicos anotados na primeira e segunda fase da coorte, nos registros de alistamento militar e nas mídias sociais. A partir dessas estratégias de busca, 687 compareceram para a coleta de dados.

Para ampliar o tamanho da amostra do estudo e prevenir perdas futuras, decidiu-se incluir outros adolescentes nascidos no município de São Luís entre 1997–1998 que não haviam sido incluídos na amostra original da coorte no momento do nascimento, realizando-se um sorteio no banco de dados do SINASC (sistema de informações sobre nascidos vivos). Uma segunda estratégia foi a inclusão de adolescentes voluntários identificados nas escolas, universidades e pelas mídias sociais. Os dados do nascimento destes adolescentes foram respondidos pela mãe. Os seguintes critérios foram levados em consideração para o cadastro: ter nascido em maternidade, na cidade de São Luís, no ano de 1997.

A partir desta listagem foi feito sorteio aleatório, obtendo-se um total de 4.593 nascidos em 1997, na cidade de São Luís. Deste total, foi possível fazer contato com 1.716, aos quais foram aplicados todos os questionários. Em uma segunda etapa, os voluntários foram identificados nas escolas, universidades e pelas mídias sociais, sendo possível fazer contato com 110. Eles foram submetidos aos mesmos testes e questionários que os demais participantes da coorte original.

Por meio dessas estratégias para inclusão de novos participantes, 1.826 adolescentes foram adicionados à pesquisa a partir da terceira fase desta coorte, a qual foi composta por 2.515 adolescentes, da original e aqueles incluídos a partir desta fase^10^.

Dessa forma, foram incluídos adolescentes nascidos em São Luís – MA, entre março de 1997 e fevereiro de 1998, que apresentaram informações acerca de dados socioeconômicos e demográficos, do nascimento e de sua saúde óssea. Não foram incluídas adolescentes grávidas, pois estas não puderam ser submetidas ao exame de densitometria óssea, uma vez que o equipamento emite uma pequena quantidade de radiação durante a avaliação, o que totalizou 2.112 adolescentes para este estudo.

### Procedimentos de Coleta de Dados e Variáveis

A coleta de dados da terceira fase ocorreu no ano de 2016. Foi realizado o preenchimento de questionários e exames de avaliação da composição corporal, por profissionais da área de saúde devidamente treinados. As informações foram registradas no programa *Research Eletronic Data Capture* (Redcap^®^) que é online e seguro para o registro e armazenamento de dados de pesquisas^11^.

Para verificar possível viés de seleção, as variáveis sexo e a mãe saber ler e escrever à época do nascimento foram comparadas entre os membros da coorte original e os adolescentes incluídos no estudo, onde pôde-se observar diferenças em direções opostas.

As perdas do acompanhamento foram maiores para as mulheres (79,0%) quando comparadas com os homens (71,5%, p < 0,001), e para aquelas de 0–4 anos de estudo (80,2%) em comparação àquelas com mais de 12 anos de estudo (74,8%, p = 0,020). Contudo, a abertura da coorte para inserção dos adolescentes trouxe maior participação de indivíduos do sexo feminino e de mulheres com menor escolaridade^10 , 12^.

### Variáveis

A variável independente explanatória principal foi o peso ao nascer, usada de forma contínua. Para os indivíduos que participaram da coorte desde o nascimento, essa variável foi coletada via prontuário nas maternidades, e, para aqueles incluídos na terceira fase, foi autorreferida pelas mães e confirmada pelo banco de dados do SINASC.

A DMO foi considerada a variável dependente do estudo. É definida pela relação entre o CMO e a área do osso avaliada, sendo expressa em g/cm^2^. A avaliação da densidade mineral óssea dos adolescentes foi realizada por meio de densitometria por dupla emissão de raios-X (DEXA), baseado em enCORE e de modelo Lunar Prodigy da marca GE Healthcare^®^. O exame é seguro e leva cerca de 15 minutos para fazer o escaneamento do corpo inteiro. Foi solicitado ao adolescente deitar-se na mesa em decúbito dorsal e permanecer imóvel durante a realização do escaneamento. O adolescente usava roupa leve, de lycra, justa ao corpo, tipicamente uma bermuda curta, para homens e mulheres, e um “top”, para as mulheres. Ficavam descalços, sem próteses dentárias e outros tipos de materiais metálicos.

Foi considerado o Z-score calculado pela diferença entre a DMO do adolescente e a média da população de mesma idade, sexo e etnia. Foi considerada a medida de corpo total, vista como um dos sítios mais sensíveis para avaliar densidade mineral óssea por estar mais associado ao maior risco de fraturas e doenças ósseas nesta população^14^. Com o objetivo de determinar as taxas de baixa DMO, esta foi categorizada em normal quando maior ou igual a -2 desvios padrões, e baixa quando menor que -2 desvios padrões^13^. A densidade mineral óssea foi utilizada como variável contínua nas análises.

Os dados maternos usados foram todos relativos à época do nascimento: idade materna (< 20 anos, 20–34 anos e ≥ 35 anos), saber ler e escrever (não/sim), situação conjugal (com/sem companheiro), paridade (um parto, dois partos e ≥ três partos) e renda familiar (até um salário mínimo, dois salários mínimos e ≥ três salários mínimos), tabagismo durante a gestação (não/sim), número de consultas realizadas durante o pré-natal (< 6 consultas e ≥ 6 consultas) e o tipo de parto (vaginal/cesárea).

Dados dos adolescentes: sexo (masculino/feminino), idade (18–19 anos), escolaridade materna (fundamental, ensino médio, curso técnico-nível médio, ensino superior incompleto e educação para jovens e adultos), situação conjugal (com/sem companheiro), classe econômica segundo o Critério de Classificação Econômica Brasil (ABEP, 2015) e atividade remunerada (sim/não).

### Modelo Teórico da Associação Entre o Peso ao Nascer e a DMO na Adolescência

Os gráficos acíclicos direcionados (DAG) são diagramas causais utilizados para selecionar variáveis, com o objetivo de controlar confundimento e evitar ajustes desnecessários. Um modelo teórico foi construído, com base na literatura, para analisar a associação entre o peso ao nascer e a DMO na adolescência ( Figura ), utilizando o *browser Dagitty* ^14^.

**Figura:** Gráfico acíclico direcionado sobre a associação do peso ao nascer e densidade mineral óssea (DMO) em adolescentes. O gráfico acíclico direcionado (DAG) foi construído com base nos modelos teóricos de Martínez-Mesa^6^e Baird^20^para predição da massa óssea. O diagrama utiliza um conjunto de setas para caracterizar associações causais entre exposição e desfecho e, além disso, identificar relações entre variáveis que influenciam a exposição ou o desfecho. Nesse tipo de gráfico, as causas são chamadas de ancestrais. Desta forma, o DAG permitiu selecionar um conjunto adequado de variáveis de confusão, bem como identificar variáveis de colisor em uma via não causal ou de polarização, a serem retidas dentro do modelo^15^. O vértice (círculo) em amarelo indica a exposição, enquanto que aquele em azul com contorno preto indica o desfecho. Os demais vértices em vermelho são ancestrais da exposição e aqueles em azul, ancestrais do desfecho. As arestas (setas) dos caminhos de polarização estão destacadas em vermelho e a do caminho causal em verde.

Na análise dos DAG foram sugeridos dois modelos: um de efeito total – onde não são incluídas as variáveis mediadoras – que sugeriu ajuste para idade materna, situação conjugal, paridade, renda familiar, realização de pré-natal, tabagismo, tipo de parto, saber ler e escrever à época do nascimento. E outro, de efeito direto – o qual são acrescidas as variáveis mediadoras, buscando-se decompor um efeito não mediado. Neste modelo analisou-se o conjunto mínimo de variáveis: paridade, renda familiar, realização de pré-natal, tabagismo e a mãe saber ler e escrever à época do nascimento.

### Análise Estatística

Os dados foram exportados do REDCap para serem analisados no programa estatístico Stata^®^versão 14.0. Para verificar se as variáveis de estudo seguiam o padrão da distribuição normal, foram construídos gráficos (histogramas) de distribuição e aplicou-se o teste de Shapiro Wilk. As variáveis categóricas foram descritas por meio de frequências absolutas e relativas. As variáveis contínuas foram descritas pela média e desvio padrão.

Foi utilizada a análise de regressão linear múltipla para testar a associação entre o peso ao nascer e a DMO na adolescência. Realizou-se, também, análise de resíduos com o objetivo de checar os pressupostos do modelo de regressão linear e identificação de *outliers.*

O nível de significância foi fixado em 5%. Estimou-se o coeficiente de regressão (Coef.) e os respectivos intervalos de confiança (IC95%).

### Aspectos Éticos

O projeto foi aprovado pelo Comitê de Ética em Pesquisa do Hospital das Clínicas da Faculdade de Medicina de Ribeirão Preto (Nº 28/2004 e Nº 10073/2009) e Comitê de Ética em Pesquisa do Hospital Universitário/UFMA (Nº 3104-476/2005). Em 2015, também aprovado pelo Comitê de Ética em Pesquisa do Hospital Universitário/UFMA (Nº 19/2015). Todos os participantes assinaram o Termo de Consentimento Livre e Esclarecido.

## RESULTADOS

Os adolescentes da coorte de São Luís tiveram prevalência de 2,8% para massa óssea baixa para a idade cronológica e o Z-escore médio de corpo inteiro foi de 0,19 (± 1,00). Na amostra de 2.112 adolescentes, 95,1% das mães não fumaram durante a gestação, 68,4% estavam na faixa etária de 20–34 anos, 95% sabiam ler e escrever, 77,5% viviam com companheiro, 49,6% eram primigestas e 87% tinham renda familiar de até um salário-mínimo na época do nascimento. Com relação ao pré-natal, 56% realizaram seis ou mais consultas de, 63,9% tiveram parto vaginal e 8,2% dos recém-nascidos apresentaram baixo peso ao nascimento ( Tabela 1 ).

**Tabela 1:** Características socioeconômicas e demográficas das mães e de seus recém-nascidos, na primeira fase da coorte de nascimento RPS. São Luís, MA, Brasil, 1997–1998.

Variáveis	n	%
Idade (anos)		
< 20	574	27,2
20–34	1.444	68,4
≥ 35	94	4,4
Saber ler e escrever		
Sim	2.009	95,0
Não	103	5,0
Situação conjugal		
Com companheiro	1.637	77,5
Sem companheiro	475	22,5
Paridade (partos)		
1	1.047	49,6
2	629	29,8
≥ 3	436	20,6
Renda familiar (SM)		
≤ 1	1.838	87,0
2	171	8,1
≥ 3	103	4,9
Tabagismo		
Não	2.007	95,1
Sim	105	4,9
Número de consultas durante o PN		
< 6	929	44,0
≥ 6	1.183	56,0
Tipo de parto		
Vaginal	1.350	63,9
Cesárea	762	36,1
Sexo do RN		
Masculino	1.008	47,7
Feminino	1.104	52,3
BPN		
Não	1.939	91,8
Sim	173	8,2

Total	2.112	100

SM: salário-mínimo; PN: pré-natal; RN: recém-nascido; BPN: baixo peso ao nascer.

Observou-se também que prevaleceram adolescentes do sexo feminino (52,3%), com 18 anos (73,7%), solteiros (96,8%), pertencentes à classe econômica C (76,2%), sem atividade remunerada (60,8%) e cursavam ensino médio (34,1%) ( Tabela 2 ).

**Tabela 2:** Características socioeconômicas e demográficas de adolescentes, na terceira fase da coorte de nascimento RPS, São Luís, MA, Brasil, 2016–2017.

Variáveis	n	%
Sexo		
Masculino	1.008	47,7
Feminino	1.104	52,3
Idade (anos)		
18	1.599	73,7
19	513	26,3
Escolaridade materna		
Fundamental	7	0,3
Ensino médio	720	34,1
Curso técnico-nível médio	125	6
Ensino superior incompleto	536	25,4
EJA	46	2,2
Ignorado	678	32
Situação conjugal		
Sem companheiro	2.045	96,8
Com companheiro	67	3,2
Classe econômica^a^		
A/B	264	12,5
C	1.056	76,2
D/E	233	11,0
Ignorado	11	0,3
Atividade remunerada		
Sim	828	39,2
Não	1.284	60,8
DMO		
Normal	2.053	97,2
Baixa	59	2,8

Total	2.112	100

EJA: Educação para Jovens e Adultos; DMO: densidade mineral óssea.

^a^De acordo com a ABEP (2015).

O peso ao nascer mostrou-se associado à DMO na adolescência (Coef.: 0,10; IC95% 0,02–0,18). Esse efeito do peso ao nascer foi atenuado na análise do modelo ajustado para os confundidores (p = 0,014; Coef.: 0,10; IC95% 0,02–0,18). Contudo, neste modelo de efeito direto, a renda familiar mais alta (p = 0,030; Coef.: -0,33; IC95% -0,66–0,33) e a mãe saber ler e escrever (p = 0,024; Coef.: 0,23; IC95% 0,03–0,43), permaneceram associados ao desfecho ( Tabela 3 ).

**Tabela 3:** Análise ajustada dos Modelos de Efeito Total e de Efeito Direto dos fatores associados ao peso ao nascer com a densidade mineral óssea (DMO) na adolescência da coorte de nascimento RPS. São Luís, MA, Brasil, 2016–2017.

Variáveis	DMO na adolescência					

	Modelo de Efeito Total			Modelo de Efeito Direto		

	Coef.	95%IC	p	Coef.	IC95%	p
Idade materna (anos)						
< 20	-0,05	-0,15 a 0,05	0,353	-	-	-
20–34	1	-	1	-	-	-
≥ 35	0,03	-0,20 a 0,26	0,772	-	-	-
Situação conjugal	0,04	-0,14 a 0,06	0,453	-	-	-
Paridade (partos)						
2	0,03	-0,07 a 0,13	0,599	0,03	-0,07 a 0,13	0,599
≥ 3	-0,08	-0,21 a 0,04	0,178	-0,08	-0,21 a 0,04	0,180
Renda familiar (SM)						
2	-0,08	-0,57 a 0,40	0,736	0,10	-0,17 a 0,36	0,480
≥ 3	-0,48	-0,85 a 0,10	**0,012**	-0,33	-0,66 a 0,33	**0,030**
Realização de PN	-0,01	-0,10 a 0,08	0,871	-0,01	-0,01 a 0,08	0,844
Tabagismo	-0,06	-0,25 a 0,14	0,570	-0,05	-0,25 a 0,14	0,583
Tipo de parto	0,01	-0,08 a 0,11	0,819	-	-	-
Saber ler e escrever	0,49	0,10 a 0,91	**0,017**	0,23	0,03 a 0,43	**0,024**
Peso ao nascer	0,10	0,02 a 0,18	**0,012**	0,10	0,02 a 0,18	**0,014**

Coef.: coeficiente de regressão; IC95%: intervalo de confiança de 95%; SM: salário mínimo; PN: pré-natal. Modelo de efeito total: ajustado para idade materna, situação conjugal, paridade, renda familiar, realização de PN, tabagismo, tipo de parto, saber ler e escrever e peso ao nascer.

Modelo de efeito direto: ajustado para paridade, renda familiar, realização de PN, tabagismo, saber ler e escrever e peso ao nascer. Somente as variáveis com p-valor < 0,05 foram consideradas estatisticamente significantes.

No modelo para efeito total (p = 0,012; Coef.: 0,10; IC95% 0,02–0,18) e direto (p = 0,014; Coef.: 0,10; IC95% 0,02–0,18) foi observada associação linear positiva entre o peso ao nascer e a massa óssea na adolescência, mesmo após ajuste para as variáveis renda familiar (p = 0,030; Coef.: -0,33; IC95% -0,66–0,33) e mães que sabiam ler e escrever (p = 0,024; Coef.: 0,23; IC95% 0,03–0,43).

Observou-se, ainda, que existe uma relação linear entre o peso ao nascer e a massa óssea na adolescência, na qual à medida que o peso ao nascer (p = 0,012; Coef.: 0,10; IC95% 0,02–0,18) aumenta em 1 kg, a massa óssea na adolescência também aumenta em 0,10 g/cm^2^. Ou seja, quanto maior o valor do peso ao nascimento, maior será o valor de DMO alcançado na adolescência ( Tabela 3 ).

## DISCUSSÃO

Neste estudo, foi observado que à medida que o peso ao nascer aumenta, os valores da DMO na adolescência também aumentam, sendo verificada associação independente do ajuste para renda familiar e a mãe saber ler e escrever à época do nascimento. A magnitude desta associação foi reduzida pelo ajuste do modelo de efeito direto para as variáveis citadas.

Poucos adolescentes apresentaram baixa densidade mineral óssea (2,8%). Ainda assim, ressalta-se que estes valores são indicativos de comprometimento da massa óssea previamente, pois aos 18 e 19 anos ocorre o pico da massa óssea, momento em que se espera que ela esteja em valores normais. Estes achados são importantes para investigação de fatores, como o desenvolvimento fetal, peso ao nascer, fatores genéticos –principais determinantes da obtenção do pico de massa óssea na vida adulta –, além de fatores modificáveis (alimentação, atividade física e tabagismo), que estão causando uma menor DMO de forma precoce^15^.

Observou-se ainda que à medida que o peso ao nascer aumenta em 1 kg, a DMO na adolescência também aumenta em 0,10 g/cm^2^, o que configura um achado relevante. Esta relação pode ser explicada pelo fato do estado de saúde do esqueleto, nas diferentes etapas da vida, ser um reflexo de etapas anteriores. Estudos prospectivos demonstram que o baixo peso ao nascer tem relação com o desenvolvimento de doenças crônicas na idade adulta, segundo a teoria Origens do Desenvolvimento da Saúde e Doenças (DOHaD)^6^.

Embora ainda não exista um consenso de como este processo acontece, uma das possíveis explicações para esta relação seria os hormônios GH (hormônio do crescimento) e cortisol estarem entre os determinantes da perda óssea^16^, ratificando a teoria de que estressores ambientais durante a vida intrauterina ou pós-natal precoce provocam alteração na sensibilidade da placa de crescimento ao GH e ao cortisol, resultando em redução do tamanho do esqueleto, podendo levar a diminuição na mineralização, e predispor a uma taxa acelerada de perda óssea durante a vida adulta^17^.

É válido ressaltar que o peso ao nascer é um importante determinante do pico de massa óssea atingido na adolescência, bem como um dos fatores principais para manutenção de níveis adequados de massa óssea na vida adulta. Apesar da existência de algumas lacunas nos mecanismos que envolvem esta relação, alguns estudos corroboram a hipótese de que a osteoporose pode ser programada no início do desenvolvimento^6 , 18 , 19^. De acordo com Baird et al.^19^, essa programação pode ser classificada em dois estudos: 1) aqueles que exploraram a relação entre o sistema fisiológico de indivíduos que podem ter sido “programados” e a taxa de perda de massa óssea devido ao envelhecimento e 2) estudos que investigaram a influência da constituição do corpo, nutrição e estilo de vida das mães sobre a massa óssea de seus descendentes.

Uma revisão sistemática^20^corroborou com os nossos achados ao mostrar que o peso ao nascer está associado à DMO, contribuindo com o conhecimento que esta associação também é observada em adolescentes. Esta é uma descoberta importante para as políticas de saúde pública, uma vez que uma redução na densidade mineral óssea está fortemente associada a um risco aumentado de fraturas^20^.

Pode-se observar ainda que, o fato de a mãe saber ler e escrever à época do nascimento e possuir uma renda familiar de, no mínimo, três salários mínimos teve efeito direto sobre a associação aqui estudada. Indivíduos expostos a condições socioeconômicas favoráveis e consequentemente estilo de vida mais saudável parecem desenvolver melhor o seu potencial de DMO adequada.

As mães que vivem em condições socioeconômicas desfavoráveis têm maiores chances de conceberem recém-nascidos com menor peso ao nascer^21^. Acredita-se que o suprimento insuficiente de nutrientes ao feto (ocasionado pela vulnerabilidade social) possa levar a adaptações das células e de seu metabolismo^22^e, portanto, influenciar os níveis basais de GH e cortisol na adolescência, por estarem envolvidos na obtenção do pico de massa óssea e predispor à maior perda óssea durante a vida adulta^23 , 24^.

Do ponto de vista clínico, considerando que a perda óssea é um processo natural e irreversível que ocorre com o envelhecimento, uma das melhores estratégias para a prevenção da osteoporose é otimizar o pico da massa óssea de um indivíduo^25^. Já do ponto de vista epidemiológico, quanto mais cedo for iniciado um procedimento preventivo, melhor será sua chance de alcançar resultados desejáveis^20^.

Estudo em Amsterdam mostrou associação positiva do peso ao nascer com a DMO do corpo todo e do quadril aos 36 anos. Os efeitos do peso ao nascer sobre a densidade mineral óssea (total, da coluna lombar e do colo de fêmur) são mantidos até idades avançadas, acima de 70 anos^25^.

Em um estudo realizado por Martínez-Mesa et al.^6^, pôde-se observar que o peso ao nascer foi o maior critério de DMO. Resultados como esses reforçam a hipótese levantada neste estudo, de que o peso ao nascer adequado pode ser importante para maximizar a massa óssea de forma precoce, sobretudo na adolescência^15 , 26^.

Dessa forma, valores baixos de peso ao nascer atuariam como marcadores de risco para densidade mineral óssea reduzida futura, uma vez que esta situação se caracteriza por baixa massa óssea e deterioração da microarquitetura do tecido ósseo, com subsequente aumento da fragilidade e susceptibilidade às fraturas^7^.

Este estudo apresentou como limitação a perda de sujeitos durante o seguimento, principalmente na terceira fase, devido dificuldades para encontrar os adolescentes, apesar da utilização de diversas estratégias de busca. Tais perdas podem ter contribuído para subestimar as associações em que estes estratos foram mais prevalentes. O peso ao nascer foi relatado pelas mães dos adolescentes incluídos no estudo, entretanto, todos os pesos de nascimento desses adolescentes foram confirmados no SINASC, sendo minimizada a possibilidade de viés de memória.

O consumo alimentar e a prática de atividade física dos adolescentes não foram incluídos no ajuste do modelo proposto, pois estas variáveis não foram identificadas pelo DAG.

Como pontos fortes, destacam-se: o desenho longitudinal deste estudo, o modelo teórico causal baseado em DAG para a identificação do conjunto mínimo de variáveis necessário para ajuste, a fim de estudar o efeito causal do peso ao nascer sobre a DMO. A avaliação da densidade mineral óssea usando DXA, que é considerado padrão ouro na adolescência, também é um importante ponto forte do estudo. Além disso, a DMO é um determinante importante da saúde óssea em idades mais avançadas e essa avaliação precoce, ainda na adolescência, foi possível neste estudo.

Ainda que a osteoporose seja uma doença mais prevalente em idosos, ela deve ser prevenida, a fim de promover a saúde óssea durante a infância e adolescência com o objetivo de garantir que a DMO atinja seu pico ótimo e se desenvolva adequadamente.

A realização de estudos que esclareçam e apontem dados ao entendimento empírico sobre o processo de aquisição da massa óssea durante a vida intrauterina, infância e adolescência até atingir o pico ósseo são de grande relevância, o que deixa claro a necessidade de que estudos em fases mais precoces sejam realizados.
